# Henry Maudsley, M.D., F.R.C.P.

**Published:** 1918-02-09

**Authors:** 


### HENRY MAUDSLEY, M.D., F.R.C.P.

### A Champion on the Side of Science.

DR. MAUDSLEY is dead, and the medical profession at large, as well as alienism in particular, have lost a great man. Few now living can remember a time when Maudsley was not regarded as a great authority, for he began at a very early age to write, and his earliest writings made their mark at once and stamped him as a leader. Men now in their seventies looked up to Maudsley when they were at College, and drew inspiration from him in their undergraduate days. He was the first man to recognise the bearing of the discoveries of Darwin, and of the trend of biological doctrine upon the functions of the brain, and especially upon the constitution of the minds of men in the sense that has recently been attached to the term temperament in these pages. He was deeply impressed with the notion that a man's character is the expression of the structural constitution of his brain, and that this in turn is determined by his heredity. Above all, he insisted that mind was the product of brain action, and founded his whole doctrine upon this. It is difficult for us in these days to realise the utter darkness that prevailed in those as to the relation not only of the brain to the mind—we know little or nothing about that now—but also of the brain to the body. Maudsley took his degree in 1856, and it was not until the experiments of Hitzig and Ferrier twenty years afterwards that the first gleam of light was thrown on the relation of the cerebral convolutions to the bodily functions. Before those investigations the convolutions were looked upon much in the light of the keys of a piano, in which the mind, as an independent and self-willed performer, played its antics. Maudsley was one of the first to recognise that the constitution of the mind, as well as the action of the mind, are strictly conditioned by brain structure.

He preached this doctrine in sonorous periods, for he was a great master of English prose. He preached with an air of great authority, as of one who had a mission to instruct and a lesson to convey. He preached not so much with enthusiasm as with deep and settled conviction, and he was extraordinarily impressive and successful. Those were days in which there was still a conflict between Science and Religion, and Maudsley was a redoubtable champion on the side of Science. Indeed, it was said of him, and not altogether without justification, that he was animated by an *odium anti-theologicum*, which was only the *odium theologicum* turned inside out. There was a certain vague but impressive grandeur in the stateliness of his denunciations; and although he taught nothing very definite, for there was no definite knowledge to teach, he roused in his many followers and admirers an enthusiasm, and inspired a conviction, that some of them retain to this day.

From 1859 to 1862 he was physician to the Manchester Royal Lunatic Asylum, a branch of the Manchester Royal Infirmary, where he had ample opportunity of studying insanity. He then migrated and returned to London—he had received his medical education at University College Hospital, and taken the degree of M.D. at the University of London—and became physician to the West London Hospital. He soon acquired a very large practice in insanity, his chief competitors being Fielding Blandford and Hack Tuke. In those days specialism as we now know it had not come into existence, nor was it established without lively controversy and much scornful opposition. The junior surgeon on the staff of a hospital lectured upon anatomy, and the assistant surgeons were demonstrators in the dissecting-room. One of the junior physicians gave lectures on physiology, and taught what little physiology there was to teach. He might even invite one or two of the more zealous students, who were going up for honours, to "look down a microscope" and see the Haversian canals of bone. There was nothing out of the way, therefore, in the appointment of a physician distinguished for his knowledge of insanity to the lectureship of medical jurisprudence at University College, and this lectureship Maudsley held for ten years. No doubt he lectured very well, for he was an extremely able man, and could master any subject that he set his mind to master. Meanwhile, books flowed rapidly from his fertile pen. Their names were various—"The Physiology of Mind," "The Pathology of Mind," "Body and Will," and so forth—but their contents were very much the same; their teaching was nebulous. And now that Ferrier had laid the basis of an anatomical connection between the convolutions and the muscles, and Hughlings-Jackson was publishing his profound and fascinating speculations, serious students wanted something more than majestic denunciations of the clergy and dithyrambic exhortations to consider the connection between bad character and a faulty constitution of the brain due to evil inheritance. From this time, and especially after a scathing review of "Body and Will," in the quarterly journal *Brain*, Maudsley's influence declined; but his mental activity did not decline, and he continued from time to time to publish more books to much the same purpose, and written in the same hortatory and prophetic style, until only two years ago, when he had completed his eightieth year. Latterly he became deaf, but he never lost interest in medicine, and especially in the treatment of the insane. His massive head and impressive handsome face were conspicuous at public meetings called to consider the treatment of mental defectives and other cognate subjects; and, as is well known, he gave to the London County Council the magnificent gift of £30,000 to found a hospital for mental diseases.

Maudsley is dead, full of years and of honours, closing in tranquillity and in general esteem and respect a life full of usefulness, crowned by a display of splendid generosity. He was little known to the later generation, but he was a power in his day, and, if he left no definite teaching behind him, he was the source of much inspiration and enthusiasm during his life.

